# Genetic variation in histidine rich proteins among Indian *Plasmodium falciparum* population: possible cause of variable sensitivity of malaria rapid diagnostic tests

**DOI:** 10.1186/1475-2875-11-298

**Published:** 2012-08-28

**Authors:** Navin Kumar, Jai PN Singh, Veena Pande, Neelima Mishra, Bina Srivastava, Ridhima Kapoor, Neena Valecha, Anupkumar R Anvikar

**Affiliations:** 1 National Institute of Malaria Research (ICMR), Sector 8, Dwarka, New Delhi, 110077, India; 2 Department of Biotechnology, Kumaun University, Nainital, Uttarakhand, India

**Keywords:** *Plasmodium falciparum* Histidine rich protein 2, *Plasmodium falciparum* Histidine rich protein 3, Rapid Diagnostic Tests, Genetic polymorphism, India

## Abstract

**Background:**

Rapid diagnostic tests (RDTs) have revolutionized the diagnosis of malaria. Among the various factors affecting RDTs sensitivity is genetic variation of the antigen used. The genetic variation in PfHRP2 and PfHRP3 proteins was studied among the Indian *Plasmodium falciparum* isolates.

**Methods:**

One hundred and forty isolates of *P. falciparum* were collected from six geographical regions of India. Target genes encoding PfHRP2 and PfHRP3 antigens were sequenced to study genetic polymorphism. Minimum detection limit giving a positive rapid diagnostic test was also determined.

**Results:**

Extensive variations were observed in amino acid repeat types of PfHRP2 and PfHRP3. PfHRP2 exhibited more polymorphism than PfHRP3. Significant relation was observed between type 2 and type 7 repeats and RDT detection rate as higher number of these repeats showed better sensitivity with RDTs.

**Conclusion:**

The results provide insights into the genetic diversity of *Pfhrp2* and *Pfhrp3* genes among Indian *P. falciparum* population and its relation to RDT sensitivity.

## Background

Malaria is an important public health problem in India with 1.5 million confirmed cases reported annually by the National Vector Borne Disease Control Programme (NVBDCP) [1]. Early diagnosis and effective treatment is one of the important strategies for malaria control and malaria RDTs form an important component of diagnosis. Detection of malaria parasites by microscopic examination of blood smears remains the gold standard but may not be easily available especially in remote areas. The development of alternative diagnostic method like RDTs has made it possible to provide rapid and accurate detection of malaria parasites in remote areas where microscopy facility is not available. RDTs are lateral immunochromatographic tests, which detect specific antigens of the malaria parasite, such as the *Plasmodium falciparum*-specific histidine rich protein 2 (PfHRP2). Other RDTs use lactate dehydrogenase and aldolase and can also detect non-falciparum infections. Most of the RDTs show excellent sensitivity and specificity for *P. falciparum* at a parasitaemia greater than 500 parasites per microlitre (parasites/μl) but at lower parasitaemia, the sensitivity is variable [2]. The majority of *P. falciparum*-specific RDTs detect *P. falciparum* histidine rich protein 2 (PfHRP2) but due to structural homology of PfHRP2 with PfHRP3, the latter also contributes to diagnosis of malaria [3].

Several factors affect the performance of malaria RDTs, which include test factors and parasite factors. But in addition to these, there is one more important factor, the variability within the parasite diagnostic antigen being detected by the RDTs. This includes presence or absence and variations in the number of the target epitopes present in a particular parasite strain. Genetic diversity may be an important factor for these antigen-based RDTs and it has been reported that the PfHRP2 antigen varies in size between different parasite strains [4,5]. The antigen consists of a number of alanine and histidine rich amino acid repeats which also affects the detection limit of RDTs [6, 7,]. Different allelic forms of these diagnostic genes have been reported in several laboratories [6-8]. However, no systematic examination of diversity in these genes has been carried out in Indian isolates, nor has any relationship between genetic diversity of diagnostic antigen and RDT detection sensitivity been examined, while variable sensitivity has been observed.

The present study was aimed to investigate the genetic diversity of *Pfhrp2* and *Pfhrp3* genes in *P. falciparum* isolates from different epidemiological strata for malaria in India as a possible cause of variable sensitivity of malaria rapid diagnostic tests.

## Methods

### Study area

**Figure 1:**
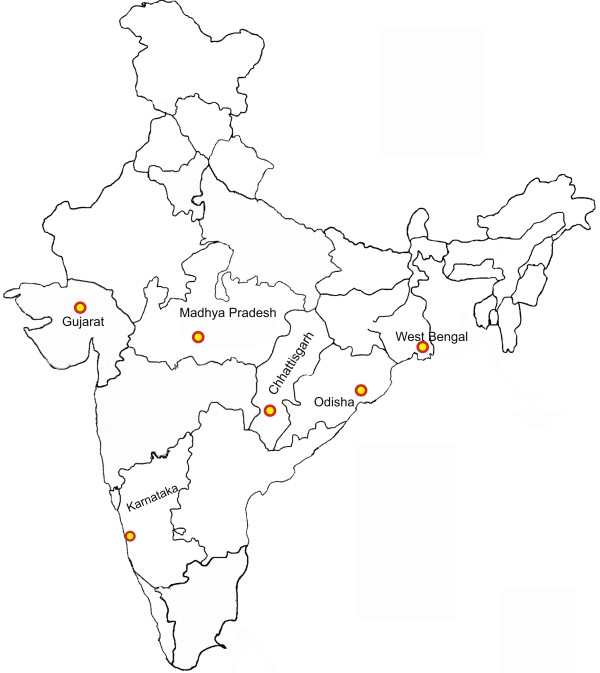
**Map of India showing geographical distribution of samples (140 *P. falciparum *isolates).1. **Chhattisgarh (n = 40), **2.** Gujarat (n = 20), **3.** Karnataka (n = 10), **4.** Madhya Pradesh (n = 20), **5.** Orissa (n = 30), **6.** West Bengal (n = 20).

Total 140 *P. falciparum* isolates were collected from six states (Chhattisgarh, West Bengal, Madhya Pradesh, Gujarat, Orissa and Karnataka) of India from December 2009 to September 2011 (Figure 1). These samples also included panels prepared for quality assurance of RDTs and culture adapted samples from the Malaria Parasite Bank of National Institute of Malaria Research (NIMR). The study was approved by institutional ethics committee of NIMR New Delhi, India. Written Informed consent was obtained from the participants. Consent was obtained from parents in case of minor. Blood samples were collected by finger prick and used for RDT testing. They were also stored as dried blood spots on filter papers. All the diagnosed cases of malaria were treated as per the national drug policy.

### RDT detection rate

Minimum parasitaemia that could be detected by RDTs was analyzed in 62 samples, which included 51 field isolates, nine quality assurance panels and two lab adapted culture lines. Parasite densities were determined against 200 white blood cells as per the WHO recommendations. Parasite blood suspensions were prepared by serial dilution with healthy human O group blood, 10, 000 p/μl, 2000 p/μl, 1000 p/μl, 500 p/μl, 250 p/μl, 125 p/μl and 62 parasites/μl. Minimum parasitaemia giving positive result was analysed with HRP2 based RDTs (Para check, Orchid Biomedical System, India). The RDT detection sensitivity was categorized as sensitive (minimum detection limit **≤** 200 parasites/μl) and non sensitive (minimum detection limit ≥ 200 parasites/μl).

### Genomic DNA isolation and diagnostic PCR

Genomic DNA was isolated from dried filter spot by QIAamp DNA mini kit (QIAGEN, California, USA) according to the manufacturer’s instructions. The identification of *P. falciparum* was confirmed by polymerase chain reaction (PCR) using species specific primers, as previously described by Snounou *et al.* at modified cycling conditions [9].

### Detection of *Pfhrp2* and *Pfhrp3* genes by PCR

Gene specific primers were used to amplify *Pfhrp2* and *Pfhrp3* genes. Two microlitres of isolated DNA was used as a template in 25 μl PCR reaction mixtures that contained 2.5 mM MgCl_2_, 10 pM of each primer and 0.2U of Amplitaq gold DNA polymerase. The DNA was initially denatured at 95°C for 5 min followed by 40 cycles of denaturation at 95°C for 30 sec, annealing at 57°C for 40 sec and extension at 72°C for 90 sec. The final extension was performed at 72°C for 10 min. Reaction cycling conditions were same for both the target genes except annealing temperature (55°C for *Pfhrp3* gene). The primer sequences as described earlier [7] were used to amplify the exon 2 of *Pfhrp2* and *Pfhrp3* genes (*Pfhrp*2 F- 5’-TGTGTAGCAAAAATGCAAAAGG-3’ *Pfhrp2* R- 5’- TTAATGGCGTAGGCAATGTG-3’, *Pfhrp3*: F- 5’-AAATAAGAGATTATTACACGAAAG-3’, *Pfhrp3*: R- 5’- TGGTGTAAGTGATGCGTAGT-3’). Ten microlitres of PCR products was separated by electrophoresis on a 1.5% agarose gel. The fragment size of PCR products was determined by comparison with molecular weight marker (100 bp) using molecular weight analysis of gel documentation system.

### Direct sequencing of PCR products and translation

All PCR products were purified by use of spin columns (QIAGEN) according to manufacturer’s instructions and were used in a standard dye-terminator DNA sequencing reaction (ABI). Nucleotide sequences were translated to corresponding amino acids and each type of amino acid repeat sequences were identified and given numeric codes from type 1 to type 18 as described in previous study [6].

### Data analysis

Sequences of poor quality were not analysed. Amino-acid sequences of the corresponding proteins were deduced from the nucleotide sequences. The predictive binary logistic regression analysis model developed by Baker *et al.* was used to evaluate isolates as sensitive (≤ 200 parasites/μl) and non sensitive (≥ 200 parasites/μl) by PfHRP2 based RDT. Bio Edit Sequence Alignment Editor Software [10] was used to align the nucleotides and deduce amino acid sequences of different isolates with the reference sequences of *P. falciparum* strains, 7 G8 for *Pfhrp2* (Accession M13986) and FCC1/HN (Accession AF202093) for *Pfhrp3*, respectively. Differences in mean number of amino acid repeats between six different regions were assessed for each type of repeats by Kruskall-Wallis test (H- test) using SPSS software. P value < 0.05 was interpreted as indicating statistically significant differences.

## Results

### Genetic variations in *Pfhrp2* and *Pfhrp3*

Of the 140 *P. falciparum* isolates, 108 samples showed PCR amplification for *Pfhrp2* fragments. These PCR amplified fragments showed extensive variations in size and ranged from 472 to 1,000 bp, while coding proteins ranged from 157 to 333 amino acids. Eight of 14 previously identified amino acids were detected [6] along with five unique repeat sequences. A total of 13 repeat type sequences of *Pfhrp2* were identified in Indian isolates. All *Pfhrp2* sequences showed a similar structure, starting with type 1 repeat (AHHAHHVAD) and ended with type 12 (AHHAA) repeat sequences except in Gujarat isolates. The central part of *Pfhrp2* sequences contained the combination of types 2, 4,5,6,7 and 8 repeat sequences. Among all 13 repeat sequences, type 2 (AHHAHHAAD) & type 7 (AHHAAD) were more frequent. Some repeats (type 1, 2, 3, 6, 7 and 11) were common in all, whereas others (type 9, 10, 12 and type 13) were not observed or were found only in few isolates. Two unique sequences type 9 (AHHAPH) and type 10 (AHHAPD) were found only in isolates from Chhattisgarh. Isolates from West Bengal showed higher frequency of type 2 repeats (46.87%) than isolates from other geographical regions of India and low frequency of type 3 repeats (3.12%) than other regions.

Variations in fragment size and amino acid repeat types were more extensive in *Pfhrp2* as compared to *Pfhrp3*. Some of the repeat types 1,2,3,6,7,11 were observed in all isolates. Number of each repeat and total number of repeats within *Pfhrp2* varied between different isolates of the country (Table 1). When it was compared between different geographical regions, differences were observed for six repeat types. Isolates of Madhya Pradesh had significantly less type 2 repeats (mean 6.3) than isolates from Karnataka (mean 9.8) and West Bengal (mean 9.8) (p = 0.85). For type 6 repeats, isolates of Madhya Pradesh (mean 0.6) had significantly fewer repeats than isolates of Orissa (mean 2.5) (p = 0.001). For type 7 repeats, isolates from Chhattisgarh (mean 0.7) and Karnataka (mean 2.9) had less repeats than isolates of Orissa (mean 3.9) (p = 0.001). Type 4 repeats were not found in isolates from Madhya Pradesh, but present in all other regions where as type 5 and type 8 were not found in Chhattisgarh isolates but interestingly they had type 9 (mean 0.3) and type 10 (mean 0.2) which were absent in isolates of other geographical regions. Type 12 repeat sequences were not observed in Gujarat isolates but type 13 was not found in isolates of Gujarat, Orissa and Karnataka.

**Table 1:** Distribution of amino acids repeat sequences of Pfhrp2 and Pfhrp3 in different regions of India

**Type of Repeat**	**Repeat Sequences in Pfhrp2/*Pfhrp3***	**Antigens observed**		**No. of individual repeats in different regions of India**						**Total Repeats**
		**Pfhrp2**	**Pfhrp3**	**Chhattisgarh**	**West Bengal**	**M.P.**	**Gujarat**	**Orissa**	**Karnataka**	
1	**AHHAHHVAD**	**+**	**-**	1-2	1-3	1-4	1-2	1-3	1-2	1-4
2	**AHHAHHAAD**	**+**	**-**	2-14	2-15	2-9	3-11	2-11	2-11	2-14
3	**AHHAHHAAY**	**+**	**-**	0-1	0-1	0-1	0-1	1-2	0-1	0-2
4	**AHH**	**+**	**-**	1-3	0-2	0	0-2	0-2	0-2	0-3
	*AHH*	**-**	**+**	0	0-1	0	0-2	0-2	0	0-2
5	**AHHAHHASD**	**+**	**-**	0	0-2	0-1	0-1	0-1	0-2	0-2
6	**AHHATD**	**+**	**-**	0-4	0-2	0-1	0-1	0-5	0-2	0-2
7	**AHHAAD**	**+**	**-**	0-1	0-2	1-2	1-6	1-5	0-3	0-4
	*AHHAAD*	**-**	**+**	0-1	0-1	1-2	0-1	1-2	0-1	0-2
8	**AHHAAY**	**+**	**-**	0	1-2	0-1	0-1	0-1	0-1	0-2
9	**AHHAPH**	**+**	**-**	0-1	0	0	0	0	0	0-1
10	**AHHAPD**	**+**	**-**	0-1	0	0	0	0	0	0-1
11	**AHVDD**	**+**	**-**	0-1	0-1	0-1	0-1	0-1	0-1	0-1
12	**AHHAA**	**+**	**-**	0-2	0-1	0-2	0	0-3	0-2	0-1
13	**AHHEAA**	**+**	**-**	0-1	0-1	0-1	0	0	0	0-1
14	*AHHAAN*	**-**	**+**	2-15	2-12	2-9	3-11	2-14	3-12	2-15
15	*AHHDGAHHDD*	**-**	**+**	1-2	1-2	1-2	1-2	1-2	1-2	1-2
16	*AHHDGAHHDG*	**-**	**+**	0-2	1-2	1-2	0-2	1-2	1-2	0-2
17	*AHHDG*	**-**	**+**	0-2	0	0	0	0	0	0-2
18	*AHHN*	**-**	**+**	0-2	0	0	0	0	0	0-2

Note: Bold letters - Pfhrp2 sequences, Italic – Pfhrp3 sequences.

Of the 140 *P. falciparum* isolates, 103 showed PCR amplification for *Pfhrp3*. The size of *Pfhrp3* amplified fragments ranged from 477 bp to 832 bp which encoded proteins of 159 to 277 amino acids. Five of the eight previously reported repeat types were identified (Table 1). All *Pfhrp3* fragments showed a similar structure i.e. starting with type 4 repeats (AHH) and ending with the combination of type 15 and type 16. Frequency of type 14 repeat (AHHAAN) sequences was higher in all the isolates (mean, 8.95) (p = 0.88) followed by type 15 and type 16 whereas type 17 (AHHDG) (mean 1.3) and type 18 (AHHN) (mean 1.1) were found only in isolates of Chhattisgarh. The difference in repeats number between different geographical regions was not statistically significant (P > 0.05).

### RDT detection rate

**Figure 2:**
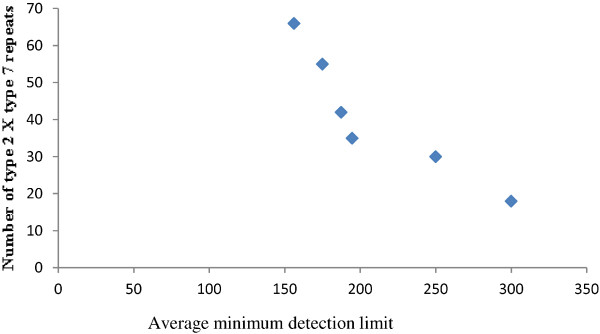
Relationship between number of type 2 × type 7 amino acid repeats of PfHRP2 and average minimum detection limit of RDTs among six different geographical regions of India.

Minimum parasitaemia that could be detected by RDTs was analysed in 62 samples***.*** Extensive variations were observed in the minimum parasite detection limit (Table 2). The minimum level of parasitaemia that could be detected by RDTs ranged from 62 p/μl to 500 p/μl (Mean- 220.7 p/μl). About 75% of the isolates were predicted to be detected by RDTs at parasite density > 200 p/μl. The relationship between type 2 × type 7 amino acid repeats of PfHRP2 and average minimum detection limit of RDTs has been presented as Figure 2. It was observed that higher number of these repeats was associated with better sensitivity of HRP2 based RDTs. The same was confirmed by Baker’s logistic regression analysis.

**Table 2:** **Distribution of 62 selected *P. falciparum *isolates/lines from different regions of India and their minimum detection limit in RDTs**

**RDTs detection limit**	**No. of *P. falciparum *isolates**							
	**Chhattisgarh**	**Madhya Pradesh**	**Gujarat**	**West Bengal**	**Orissa**	**Karnataka**	**Malaria Parasite Bank**	**Total**
62 p/μl	4	0	0	0	0	3	0	**7**
125 p/μl	10	2	3	2	8	9	2	**36**
250 p/μl	3	1	1	1	1	2	0	**9**
500 p/μl	3	2	0	1	1	3	0	**10**
**Total**	**20**	**5**	**4**	**4**	**10**	**17**	**2**	**62**

**(**Individual detection limits are given as Additional file 1: Table S1).

## Discussion

Malaria diagnostic tests provide early and accurate diagnosis of malaria. This enables prompt treatment and its control especially in remote inaccessible areas. Apart from various other factors genetic polymorphism in diagnostic antigens of *P. falciparum,* i.e. *Pfhrp2* and *Pfhrp3,* is also an important factor responsible for variable sensitivity of malaria RDTs. These antigens consist of a number of alanine- and histidine-rich amino acid repeats [6-8] and vary in size between parasite strains and comparison of the PfHRP2 sequences from several parasite strains has shown differences in the number of tri- and hexapeptide repeat units and rare amino acid variants [6]. Effects of sequence variation in *P. falciparum* histidine- rich protein 2 on binding of specific monoclonal antibodies have been also reported [11]. However, the degree of antigen variation has recently been shown to be limited for pLDH and parasite aldolase [12,13].

Only six out of the 13 identified different amino acid repeats were found to be present in all isolates. Rest of the repeats were distributed randomly among isolates from different geographical areas. Most of the RDTs for falciparum malaria are based on *P. falciparum*-specific histidine rich protein specific monoclonal antibody. If RDTs recognizes epitopes present in repeat sequences that are not universally present, it may lead to misdiagnosis of malaria or variable performance of RDTs especially at low parasitaemia. Relation between the number of type 2 and type 7 repeats and RDT detection rate was confirmed statistically. This suggests that the epitopes recognized by PFHRP2-specific monoclonal antibody reside within or near around type 2 and type 7 repeats. These two repeat types were more common and frequent in all geographical isolates of India. Similar results were also observed in global isolates [6-8].

Number of these repeats varied from 2 to 15 in *Pfhrp2* and 1 to 6 in *Pfhrp3*. The data of the present study supports the findings reported earlier. RDTs may fail to detect the isolates with less number of types 2 and 7 repeat sequences at lower parasite density i.e. ≤ 200 parasites/μl. Another possible factor affecting the sensitivity of HRP2-detecting RDTs is failure of the parasite to express the antigen, due to deletion of genes or frame shift mutations, or alterations in protein expression. However, parasite lines lacking either or both *Pfhrp2* or *Pfhrp3* genes have only been identified following laboratory adaptation to *in vitro* culture [14] and in the progeny of a genetic cross [15] now it has been characterized that a number of *P. falciparum* isolates collected in Iquitos, Peru and brazil from human patients lacked one or both of these genes that were positive by microscopy but tested negative with HRP2-detecting RDTs [16,17].

It has been reported that due to structural homology of PfHRP3 with PfHRP2, it cross reacts with antibody coated in RDTs for PfHRP2. Hence, PfHRP3 also contributes to the diagnosis of falciparum malaria [3]. The results showed that two types of amino acid repeat sequences i.e. type 4 & type 7 were common in both *Pfhrp2* and *Pfhrp3* genes. Five repeats were found only in *Pfhrp3* gene (type 14, type 15, type 16, type 17 and type 18). This suggests that some of the epitopes detected by monoclonal antibody of RDTs were also found in PfHRP3 although PfHRP3 was less variable than PfHRP2. Cross-reactivity between PfHRP2 and PfHRP3 has also been reported for several PfHRP2-specific monoclonal antibodies like 2 G12, MAB87 and ID6 [4]. The antibodies generated against PfHRP2 and PfHRP3 predominantly recognize the corresponding antigen, but weakly recognize other antigens [18]. The cross reactivity between these two antigen is also likely to modulate the effect of genetic diversity of *Pfhrp2* and *Pfhrp3* on RDTs performance. There is some published information on specific epitopes of antibodies used in RDTs [12]. Six monoclonal antibodies are also published that are directed against PfHRP2 [19-22], but due to the limited supply of monoclonal antibody, most of the PfHRP2-based RDTs use similar or identical antibodies. Hence, the effect of genetic variation in these diagnostic genes on performance of RDTs is also applicable for most of the commercial PfHRP2-based RDTs. The data predicts that ~ 25% parasites of India are not detectable at parasitaemia level < 200 parasites/μl. However, more comprehensive genetic variation analysis with larger sample size for each geographical malaria endemic region of country is needed to support this hypothesis. During this study, two cases of *Pfhrp2/Pfhrp3* gene deletion in *P. falciparum* field isolates from Chhattisgarh have also been found and will be presented as a separate report. Hence, there is need to investigate the geographical extent of Indian *P. falciparum* isolates lacking *Pfhrp2* and/or *Pfhrp3* genes. Besides this, comprehensive analysis of other target antigens of malaria RDTs, such as pLDH and aldolase among Indian isolates is also needed.

## Conclusion

The study provides Indian data on variation and sequence information of *Pfhrp2* and *Pfhrp3* genes and its effect on performance of RDTs. The results will provide platform in designing more sensitive RDTs.

### Nucleotide sequence accession numbers

The nucleotide sequences reported here have been deposited in the GenBank database under accession numbers [GenBank: JN794048, GenBank: JN794050 to GenBank: JN794053] for *P. falciparum* hrp2 sequences and [GenBank: JN794049, GenBank: JN794054 and GenBank: JN794055] for *P. falciparum* hrp3 sequences.

## Abbreviations

RDTs: Rapid Diagnostic Tests; HRP-2: Histidine Rich Proteins-2; PCR: Polymerase Chain Reaction; WHO: World Health Organization; ACT: Artemisinin based Combination Therapy; CQ: Chloroquine; NVBDCP: National Vector Borne Disease Control Programme; SPSS: Statistical Package of Social Services; CHC: Community Health Centre; PHC: Primary Health Centre.

## Competing interests

Authors declare that they have no any competing interests.

## Author’s contributions

NK, JPNS, RK, BS performed experimental work, data analysis and manuscript writing. VP and NM gave constructive advice and review the manuscript. NV and AA were involved in all stages of this study. All authors have read and approved the final version of manuscript.

## Supplementary Material

Additional file 1 **Table S1**. Minimum parasite densities among *P. falciparum* isolates/lines from different regions of India. Click here for file
