# Supplementary material for: Genetic variation in histidine rich proteins among Indian Plasmodium falciparum population: possible cause of variable sensitivity of malaria rapid diagnostic tests
Source: Malar J. 2012 Aug 28;11:298. doi: 10.1186/1475-2875-11-298 (PMC3475030; doi:10.1186/1475-2875-11-298)
Supplement: Additional file 1 — Table S1. Minimum parasite densities among P. falciparum isolates/lines from different regions of India. [file 1475-2875-11-298-S1.docx]

**Additional Table**: Minimum parasite densities among *P. falciparum* isolates/lines from different regions of India

| Regions | Study sites/Origin | Isolates/lines | Minimum detection limit |
| --- | --- | --- | --- |
|  |  |  | Paracheck |
| Field Isolates | | | |
| Central | Chhattisgarh | CBB 2 | 125 |
|  |  | CBB 3 | 62 |
|  |  | CBB 4 | 125 |
|  |  | CBB 5 | 125 |
|  |  | CBB 6 | 62 |
|  |  | CBB 7 | 125 |
|  |  | CBB 8 | 125 |
|  |  | CBB 9 | 62 |
|  |  | CBB 10 | 500 |
|  |  | CBB 25 | 500 |
|  |  | CBB 26 | 125 |
|  |  | CBB 27 | 500 |
|  |  | CBB 28 | 500 |
|  |  | CBB 29 | 125 |
|  |  | CBB 30 | 125 |
|  |  | CBB 31 | 125 |
|  |  | CBB 33 | 500 |
|  |  | CBB 35 | 62 |
|  |  | CBB 38 | 125 |
|  |  | CBB 44 | 250 |
|  | Madhya Pradesh | BH 4A | 500 |
|  |  | BH 7A | 500 |
|  |  | BH 1B | 250 |
|  |  | BH 4B | 250 |
|  |  | BH 7B | 500 |
| West | Gujarat | GJ4/217 | 250 |
|  |  | GJ3/11 | 125 |
|  |  | GJ2/94 | 125 |
|  |  | GA 50 | 125 |
| East Central | West Bengal | NK 44 | 250 |
|  |  | NK 48 | 125 |
|  |  | NK 50 | 125 |
|  |  | NK 52 | 500 |
|  | Orissa | T 2 | 500 |
|  |  | T 4 | 125 |
|  |  | T 6 | 125 |
|  |  | T 8 | 125 |
|  |  | OC 50 | 125 |
|  |  | OC 52 | 250 |
|  |  | OC 54 | 125 |
|  |  | OC 56 | 125 |
| South | Karnataka | KM 2 | 125 |
|  |  | KM 4 | 125 |
|  |  | KM 6 | 125 |
|  |  | KM 7 | 250 |
|  |  | KM 8 | 125 |
|  |  | KM 9 | 250 |
|  |  | KM 10 | 62 |
|  |  | KM 11 | 125 |
|  |  | KM 20 | 500 |
|  |  | KM 22 | 125 |
| QA panels | | | |
| East Central | Orissa | RKL 1941 | 125 |
|  |  | RKL 1966 | 125 |
| South | Karnataka | M 1 | 500 |
|  |  | M 2 | 125 |
|  |  | M 3 | 62 |
|  |  | M 4 | 125 |
|  |  | M 5 | 500 |
|  |  | M 6 | 125 |
|  |  | M 7 | 62 |
| Cultured lines | | | |
| New Delhi | Malaria Parasite Bank | MPB 1 | 250 |
|  |  | MPB 2 | 500 |
|  | **Total** | **62** |  |
